# Impact of maternal cigarette smoke exposure on brain inflammation and oxidative stress in male mice offspring

**DOI:** 10.1038/srep25881

**Published:** 2016-05-12

**Authors:** Yik Lung Chan, Sonia Saad, Carol Pollock, Brian Oliver, Ibrahim Al-Odat, Amgad A. Zaky, Nicole Jones, Hui Chen

**Affiliations:** 1 School of Life Sciences, Faculty of Science, University of Technology Sydney, Broadway, NSW 2007 Australia; 2 Renal group, Kolling Institute of Medical Research, Royal North Shore Hospital, the University of Sydney, NSW, 2065 Australia; 3 Department of Pharmacology, School of Medical Sciences, University of New South Wales, NSW 2051, Australia

## Abstract

Maternal cigarette smoke exposure (SE) during gestation can cause lifelong adverse effects in the offspring’s brain. Several factors may contribute including inflammation, oxidative stress and hypoxia, whose changes in the developing brain are unknown. Female Balb/c mice were exposed to cigarette smoke prior to mating, during gestation and lactation. Male offspring were studied at postnatal day (P) 1, P20 and 13 weeks (W13). SE dams had reduced inflammatory mediators (IL-1β, IL-6 and toll like receptor (TLR)4 mRNA), antioxidant (manganese superoxide dismutase (MnSOD)), and increased mitochondrial activities (OXPHOS-I, III and V) and protein damage marker nitrotyrosine. Brain hypoxia-inducible factor (HIF)1α and its upstream signalling molecule early growth response factor (EGR)1 were not changed in the SE dams. In the SE offspring, brain IL-1R, IL-6 and TLR4 mRNA were increased at W13. The translocase of outer mitochondrial membrane, and MnSOD were reduced at W13 with higher nitrotyrosine staining. HIF-1α was also increased at W13, although EGR1 was only reduced at P1. In conclusion, maternal SE increased markers of hypoxia and oxidative stress with mitochondrial dysfunction and cell damage in both dams and offspring, and upregulated inflammatory markers in offspring, which may render SE dams and their offspring vulnerable to additional brain insults.

Cigarette smoking is a significant risk factor for a number of chronic conditions, such as cerebrovascular and cardiovascular diseases, in addition to respiratory disorders^1^, and thus remains a major cause of death worldwide^2^. Despite general education on the risks, smoking during pregnancy and passive smoking during pregnancy are still common in both developed and developing countries^3^,^4^, and ~20–45% women smoke during pregnancy in Europe, Australia, South America, and South Africa^3^,^4^,^5^. Smoking and second hand smoking in pregnant women may result in placental transfer of toxic agents present in cigarettes and transmit a risk to the developing fetal brain. In addition there are increased risks of developing well-known metabolic, respiratory and behavioural disorders that are recognised in the offspring of first-hand or second-hand smoking mothers (reviewed in^6^,^7^,^8^,^9^). Nicotine can pass through the placenta and act as a vasoconstrictor, which can reduce uterine blood flow by up to 38%^10^, leading to deprivation of oxygen and nutrients in the fetus, resulting in hypoxia and undernutrition^11^. As such, maternal smoking is a known risk factor for intrauterine growth retardation^12^,^13^, with adaptive brain structural and functional changes occurring during fetal development^14^,^15^,^16^,^17^,^18^. Preterm infants from smoking mothers display significantly smaller frontal lobe and cerebellar volumes after adjustments of confounding factors such as alcohol consumption^19^. It is likely that maternal smoking alters fetal brain immune function and mitochondrial activity that make such offspring more vulnerable to brain insults.

Oxidative stress is integral to the general inflammatory response^20^, which occurs due to a metabolic imbalance brought about by excess production of reactive oxygen species (ROS, such as the superoxide anion) and/or a reduced level of host antioxidant defences. Mitochondria are a major site of ROS production during oxidative phosphorylation (OXPHOS) to generate ATP^21^. During an inflammatory response, there is a high consumption of oxygen and release of the superoxide free radical (O^−^_2_) by the mitochondria^22^, which can, in turn, impair mitochondrial function^23^ leading to cell and organ impairment. Thus, to protect cell integrity, excessive ROS are removed by antioxidants, including mitochondrial manganese superoxide dismutase (MnSOD). Oxidative stress can also exacerbate associated inflammatory reactions by activating pathways such as c-jun N-terminal kinases and nuclear factor-κ-light-chain-enhancer of activated B cells^24^. Hence, increased antioxidant levels or activity can significantly reduce the injury size in mice following stroke^25^. However, if the brain has pre-existing oxidative stress and inflammation, both mitochondrial and cellular function can be affected especially during post-injury repair^26^,^27^. Cigarette smoke itself contains a substantial amount of ROS^28^, which may exceed the baseline antioxidative capacity of the mitochondria to clear both endogenous and exogenous ROS. Indeed, it has been shown that smokers have decreased levels of antioxidants in their serum^29^. However, it is unclear whether smoking increases brain inflammation and oxidative stress. Therefore, we hypothesise that there may be a causal link between cigarette smoke exposure (SE), increased inflammation, oxidative stress and mitochondrial dysfunction in the brain. The aim of this study was to investigate the impact of continuous maternal cigarette smoke exposure in mice on brain inflammation, mitochondrial function and antioxidant capacity, as well as markers of hypoxia in both mothers and offspring.

## Materials and Methods

### Maternal cigarette smoke exposure

The animal experiments were approved by the Animal Care and Ethics Committee at the University of Technology Sydney (ACEC#2011-313A). All protocols were performed according to the Australian National Health & Medical Research Council Guide for the Care and Use of Laboratory Animals. Virgin female Balb/c mice (6 weeks, Animal Resources Centre, Perth, Australia) were housed at 20 ± 2 °C and maintained on a 12-h light, 12-h dark cycle (lights on at 06:00 h) with ad libitum access to standard laboratory chow and water. After the acclimatisation period, mice were assigned to cigarette SE or sham exposure (SHAM). The SE group was exposed to 2 cigarettes (Winfield Red, ≤16 mg tar, ≤1.2 mg nicotine, and ≤15 mg of CO; VIC, Australia) in a perspex chamber (18 L), twice daily for six weeks prior to mating, during gestation and lactation; while the SHAM group was exposed to normal air as previously described^30^. They were mated with male Balb/c mice (8 weeks) from the same source, which were not exposed to cigarette smoke. The offspring were housed 4–5/cage after weaning, and the males were studied at postnatal day (P)1, P20 (weaning), and week 13. The females will be reported separately.

### Sample collection

Animals at P1 were sacrificed by decapitation, while animals older than 20 days were killed after anaesthetic overdose (Pentothal^®^, 0.1 mg/g, i.p., Abbott Australasia Pty. Ltd., NSW, Australia) between 9:00–12:00 h. The mothers were also culled between 9:00–12:00 h (with their last cigarette being at 15:00 h the previous day). Brains were dissected into the left and right hemispheres. The left hemisphere was stored at −80 °C for mRNA and protein analysis, while the right hemisphere was fixed with 4% formalin for immunohistochemical analysis.

### Quantitative real-time PCR

Total mRNA was extracted from brain tissues using TriZol reagent (Life Technologies, CA, USA). The purified total RNA was used as a template to generate first-strand cDNA using M-MLV Reverse Transcriptase, RNase H, Point Mutant Kit (Promega, Madison, WI, USA)^31^. Genes of interest were measured using manufacturer pre-optimized and validated TaqMan primers and probes (Life Technologies, CA, USA). Only the probe sequence is provided by the manufacturer (Table 1). The probes of the target genes were labelled with FAM^®^ dye and those for housekeeping 18 s rRNA were labelled with VIC^®^ dye. Gene expression was standardized to 18 s RNA. The average expression of the control group was assigned as the calibrator against which all other samples are expressed as fold difference.

### Western Blotting

The protein levels of early growth response factor (EGR)1, hypoxia-inducible factor (HIF)-1α, manganese superoxide dismutase (MnSOD), translocase of outer membrane (TOM)20 and OXPHOS complex proteins were measured by western blotting. The brain was homogenised using cell lysis buffers for whole protein and mitochondria protein extraction according to manufacturer’s instruction^32^. Protein samples (40 μg) were separated on NuPage^®^ Novex^®^ 4–12% Bis-Tris gels (Life Technologies, CA, USA) and then transferred to PVDF membranes (Rockford, IL, USA), which were blocked with non-fat milk powder and incubated with the primary antibodies (EGC-1 (1:5000, Santa Cruz Biotechnology), HIF-1α (1:1000, Novus Biologicals); MnSOD (1:1000) & TOM20 (1:2000, Santa Cruz Biotechnology), Mitoprofile Total^®^ OXPHOS complex Rodent WB antibody (1:2500, Abcam)) for overnight and then secondary antibodies (1:2000 for HIF-1α; 1:5000 for MnSOD, TOM20 and OXPHOS complex, goat anti-rabbit or rabbit anti-mouse IgG horseradish peroxidase-conjugated secondary antibody (Santa Cruz Biotechnology)) for 1 hour. Protein expression was detected by SuperSignal West Pico Chemiluminescent substrate (Thermo, MA, USA) by exposure of the membrane in FujiFilm (Fujifilm, Tokyo, Japan). Protein band density was determined using Image J software (National Institute of Health, Bethesda, Maryland, USA).

### Immunohistochemistry

Formalin fixed brain samples were embedded in paraffin and sectioned (4 μm). Three coronal sections were used from SHAM and SE respectively. They were incubated with primary antibodies against nitrotyrosine (1:400 dilution, Upstate Biotechnology, Temecula, CA) followed by horseradish peroxidase anti-rabbit Envision system (Dako Cytochemistry, Tokyo, Japan). The sections were then counterstained with haematoxylin. Three images of cortex from each section were captured and used for analysis. Calculation of the proportion of area stained positive for nitrotyrosine was then determined using Image J software (National Institute of Health, Bethesda, Maryland, USA). To confirm the antibody specificity, anti-Nitrotyrosine antibody was pre-incubated with 10 mM Nitrotyrosine in PBS for 1 h at room temperature before incubation on the tissue. This yielded no staining (data not shown).

### Statistical methods

Results are expressed as mean ± S.E.M. The difference between groups was analysed using unpaired Student *t* tests (Statistica 9, Statsoft, USA).

## Results

### Effects of cigarette smoke exposure on the dams

#### Body parameters

Both SHAM and SE dams had a similar body weight at baseline (17.8 ± 0.2 vs 17.7 ± 0.2 g, n = 10). Before mating, SHAM dams were significantly heavier than the SE dams (18.7 g ± 0.3 vs 16.8 g ± 0.2 g, P < 0.05). When pups were weaned at P20, SE dams (21.9 ± 0.2 g) were 12% lighter than the SHAM dams (24.6 ± 0.4 g, P < 0.01), who also had much higher circulating levels of cotinine, which is a metabolite of nicotine (96.5 ± 33.7 vs. 1.52 ± 0.40 ng/ml in the SHAM, P < 0.05).

#### Brain inflammatory markers

**Figure 1:**
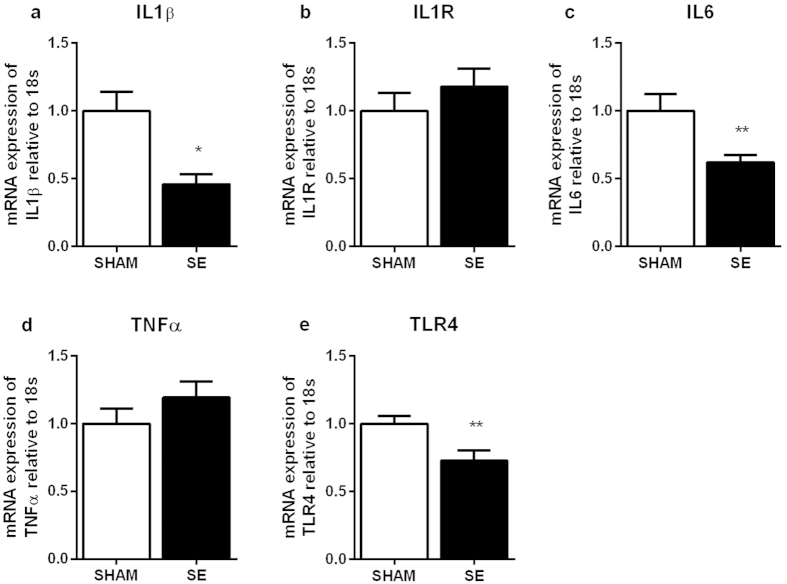
Brain mRNA expression of inflammatory markers in the SHAM and SE dams (n = 8). Results are expressed as mean ± S.E.M. Data were analysed by student’s unpaired t-test. *P < 0.05; **P < 0.01. SE: smoke exposed.

**Figure 2:**
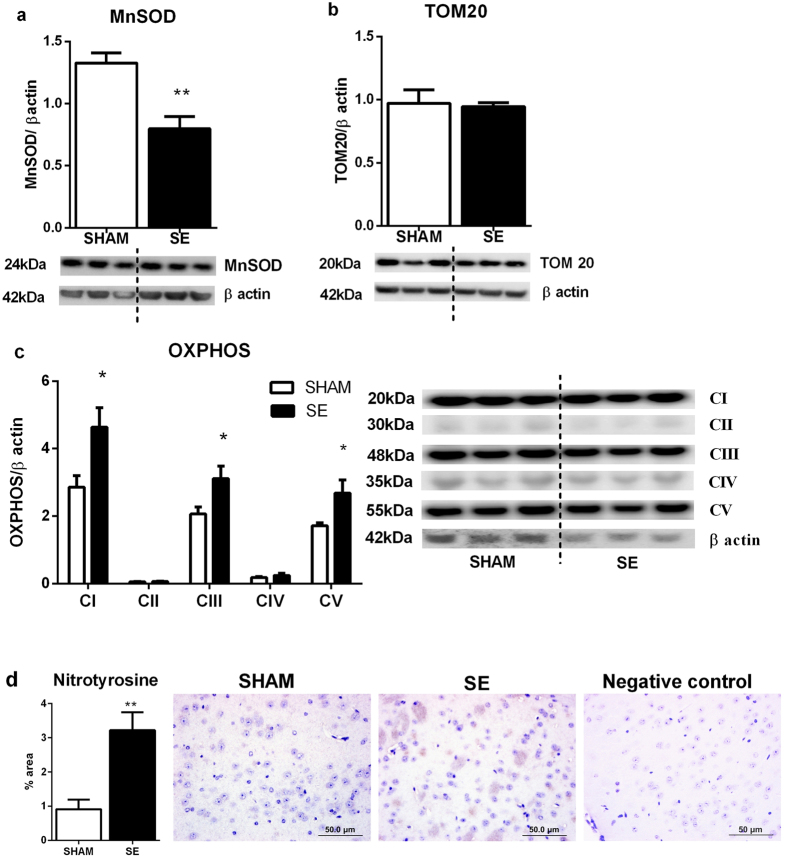
Brain protein levels of MnSOD (a), TOM20 (b) and OXPHOS complexes (CI, CII, CIII, CIV and CV) (c) in the SHAM and SE dams. Whole gel images of (**a**–**c**) in Supplementary Fig. 1. Immunohistochemistry for cortex nitrotyrosine in the dams (**d**) Scale bar = 50 μm (n = 4). Results are expressed as mean ± S.E.M. Data were analysed by student’s unpaired t-test. *P < 0.05; **P < 0.01. MnSOD: manganese superoxide dismutase; OXPHOS: oxidative phosphorylation; SE: smoke exposed; TOM20: translocase of the mitochondrial outer membrane.

Brain IL-1β, IL-6 and toll like receptor (TLR)4 mRNA expression were significantly decreased in the SE dams compared with the SHAM dams (P < 0.05, Fig. 1a; P < 0.01, Fig. 2c,e, respectively, n = 6 − 8). The expression of IL-1R and TNF-α mRNA were not different between the groups (Fig. 1).

#### Oxidative stress markers in the brain mitochondria

Brain mitochondrial MnSOD protein was reduced in the SE mothers (P < 0.01, Fig. 2a, n = 6). TOM20 protein was not different following SE. The protein levels of OXPHOS complexes CI, CIII and CV were significantly higher in the SE mothers compared to SHAM. Brain mitochondrial levels of CII and CIV were very low compared with other members of OXPHOS complexes in both SHAM and SE mothers (Fig. 2c). There was only minimal staining for nitrotyrosine in brains from SHAM mothers, and the amount and intensity of staining was greater in the SE mothers. We measured the proportion of area stained positive for nitrotyrosine and this was significantly higher in the SE group (P < 0.01, Fig. 2d). Negative IgG was performed to confirm staining specificity.

#### Brain hypoxia markers

**Figure 3:**
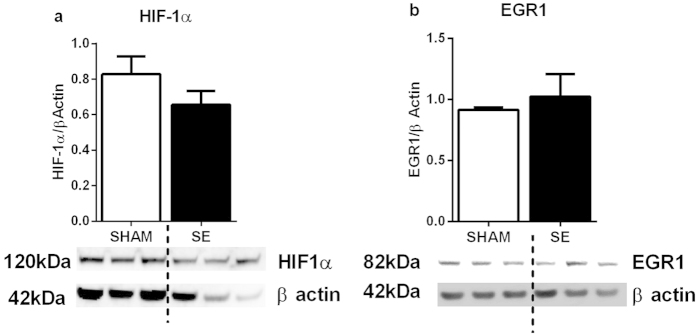
Brain protein levels of HIF-1α (a) and EGR1 (b) in the SHAM and SE dams (n = 3). Whole gel images of (**a**,**b**) in Supplementary Fig. 2. Results are expressed as mean ± S.E.M. Data were analysed by student’s unpaired t-test. EGR1: early growth response factor: HIF-1α: hypoxia-inducible factor; SE: smoke exposed.

HIF-1α protein was reduced by 20% (P = NS) in the brains from the SE mothers (Fig. 3a); while its upstream regulator EGR-1 was simular between the groups (Fig. 3b).

### Effects of maternal SE on male offspring

#### Growth

Body weights were not different between the SHAM and SE male offspring at P1 and P20 (Table 2). When the pups reached adulthood at week 13, SE offspring were significantly lighter than the SHAM offspring (P < 0.01, Table 2). Brain weights were smaller in the SE offspring at P1 and week 13 (P < 0.01), however the differences disappeared when expressed as a percentage of body weight (Table 2). Plasma cotinine levels in the SE offspring (7.60 ± 0.33 ng/ml) were double that of the SHAM offspring (3.07 ± 0.10 ng/ml, P < 0.01) at P20.

#### Brain inflammatory markers

**Figure 4:**
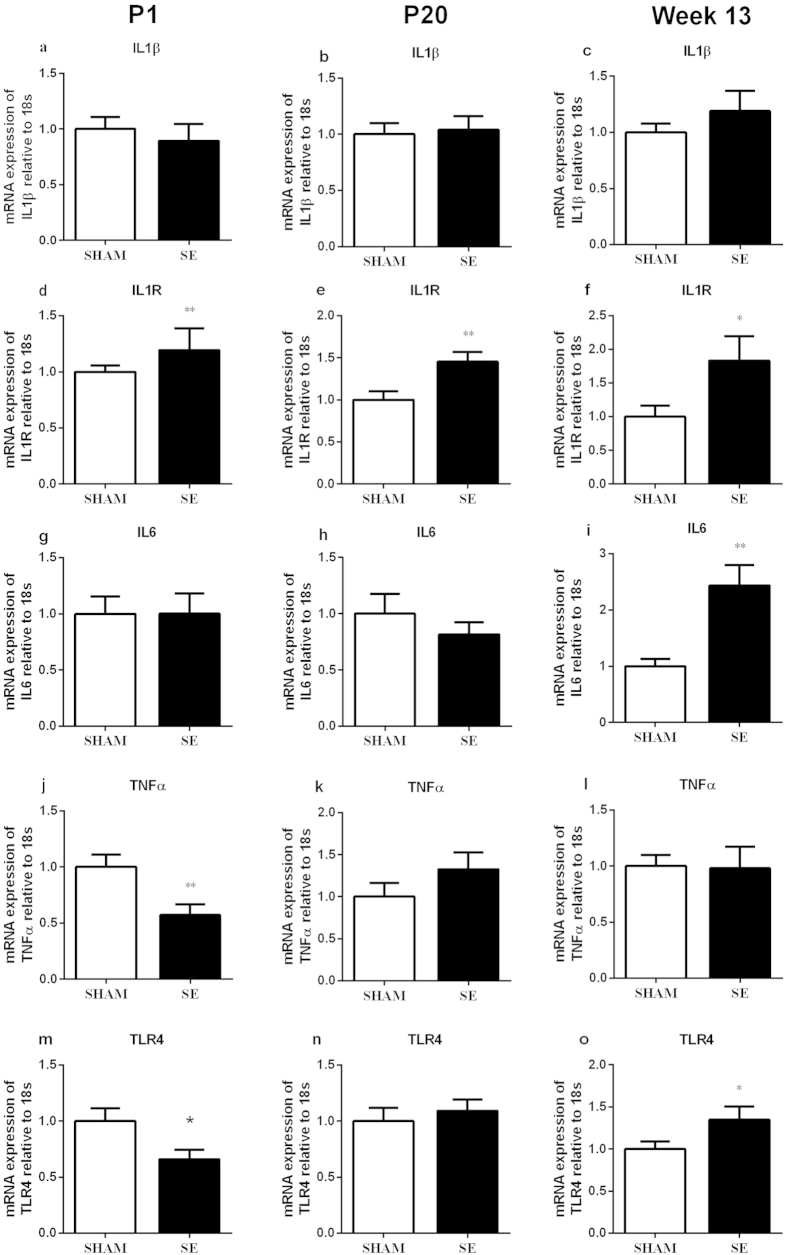
Brain mRNA expression of inflammatory markers in the offspring of SHAM and SE mothers at different ages (n = 8). Results are expressed as mean ± S.E.M. Data were analysed by student’s unpaired t-test. *P < 0.05; **P < 0.01. SE: smoke exposed.

Brain IL-1β mRNA expression was similar between groups at all ages (Fig. 4a–c). IL-1R mRNA expression was significantly increased in the SE offspring at all ages (Fig. 4d,e, P < 0.01; 4f, P < 0.05). IL-6 mRNA was upregulated in the SE offspring only at week 13 (Fig. 4i, P < 0.01). TNFα mRNA expression in the SE offspring was lower at P1 (Fig. 4j, P < 0.05), but not changed at P20 and week 13 (Fig. 4k,l) in comparison to SHAM controls. TLR-4 mRNA expression in the SE offspring was significantly decreased at P1 but increased at week 13 (Fig. 4m,j, P < 0.05) without any change at P20.

#### Oxidative stress markers in the brain mitochondria

**Figure 5:**
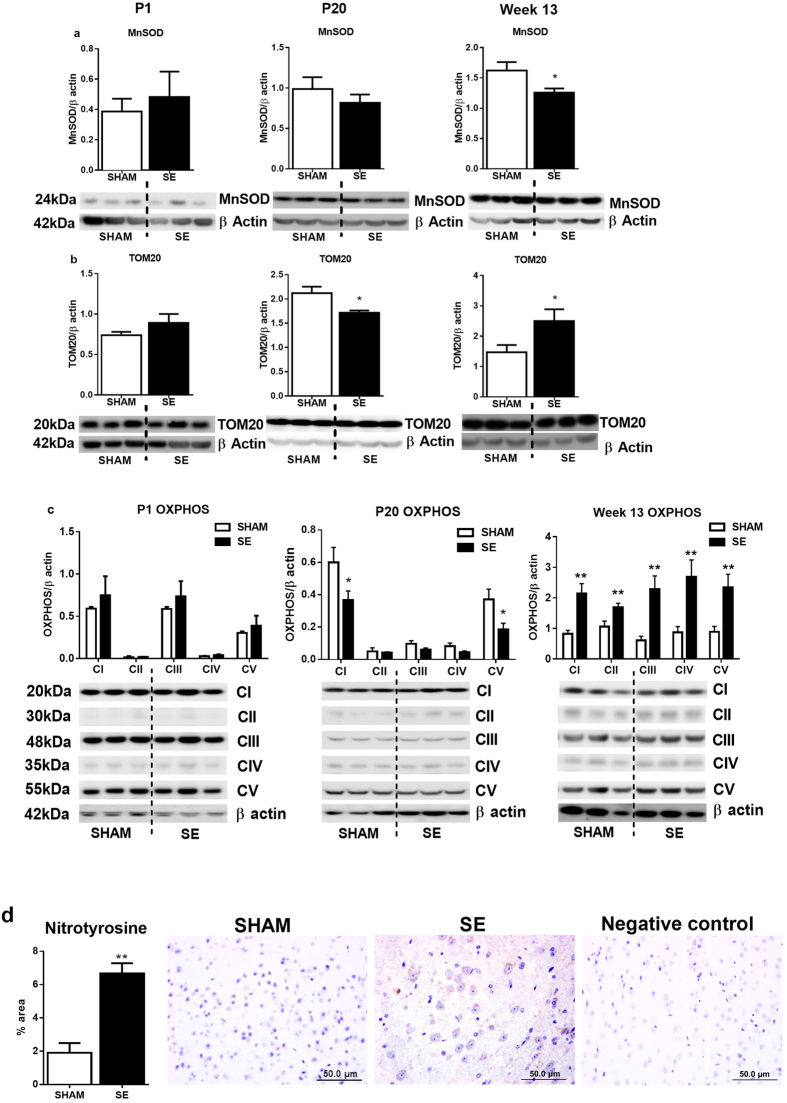
Protein expression of MnSOD (a), TOM20 (b) and OXPHOS complexes (CI, CII, CIII, CIV and CV) (c) in the brain mitochondria in the offspring of SHAM and SE mothers at different ages (n = 3). Whole gel images of (**a**–**c**) in Supplementary Fig. 3. Immunohistochemistry of cortex nitrotyrosine in week 13 offspring (**d**) Scale bar = 50 μm (n = 3). Results are expressed as mean ± S.E.M. Data were analysed by student’s unpaired t-test. *P < 0.05; **P < 0.01. MnSOD: manganese superoxide dismutase; OXPHOS: oxidative phosphorylation; SE: smoke exposed; TOM20: translocase of the mitochondrial outer membrane.

**Figure 6:**
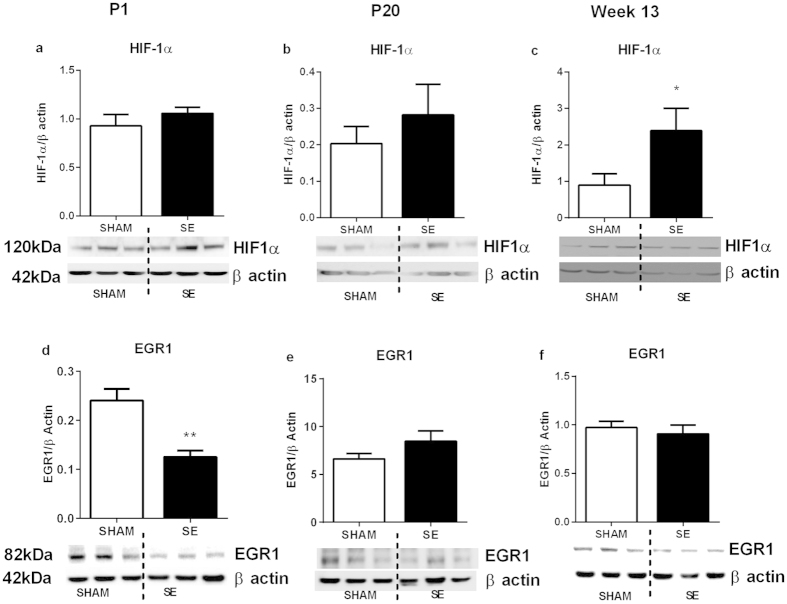
Brain protein levels of hypoxia markers in the offspring of SHAM and SE mothers at different ages (a–f) (n = 3). Whole gel images of (**a**–**c**) in Supplementary Fig. 4. Results are expressed as mean ± S.E.M. Data were analysed by student’s unpaired t-test. **P < 0.01. EGR1: Early growth response factor; HIF-1α: hypoxia-inducible factor; SE: smoke exposed.

At P1, mitochondrial protein levels of both MnSOD and TOM20 were similar between the SHAM and SE offspring (Fig. 5a,d). TOM20 protein was reduced at P20 in the SE offspring, but increased at week 13 (Fig. 6b, P <  0.05). MnSOD levels in SE offspring were reduced at week 13 (Fig. 5c,f, P < 0.05) compared to SHAM controls. OXPHOS complexes CI-V were not different between groups at P1 (Fig. 5f). At P20, brain OXPHOS CI and CV were significantly decreased in the SE offspring (Fig. 5g,P < 0.05); all the OXPHOS complexes CI–V were significantly increased in the SE offspring at week 13 (Fig. 5h,P < 0.01). Brain nitrotyrosine levels were increased in SE offspring at week 13 (Fig. 5d, P < 0.01).

#### Brain hypoxia markers

HIF-1α protein was significantly increased at week 13 in the brains of SE offspring (P < 0.05, Fig. 6c); EGR-1 was significantly reduced at P1 (P < 0.01, Fig. 6d), while unchanged at P20 and week13 in the brains of SE offspring in comparison to the offspring from SHAM mothers (Fig. 6e,f).

## Discussion

Smoking during pregnancy is considered a major and significant public health issue. A rodent model is commonly used to study the detrimental impact of maternal tobacco exposure on offspring^19^,^33^. Administration of nicotine alone is insufficient to reflect the complexity of the cigarette smoke which comprises approximately 3800 constituents^33^. Here, we have investigated the impact of maternal cigarette smoke exposure on brain inflammatory markers, oxidative stress related mitochondrial activity, and markers of hypoxia in both dams and male offspring. There were similar brain changes in both SE mothers and offspring, with respect to reduced anti-oxidative capacity of the brain, which may reduce the ability of mitochondria to scavenge excess ROS generated during increased OXPHOS activity. This is reflected by increased nitrotyrosine levels, a direct product of oxidative stress, in the brains of SE mothers. However, SE mothers and adult SE offspring had quite distinct changes, in markers of brain inflammation and hypoxia, which were lower in the SE mothers, however higher in mature SE offspring. Increased brain oxidative stress and chronic inflammation are evident in certain neurodegenerative diseases, as neurons in the brain are highly sensitive to oxidative stress^34^,^35^,^36^,^37^. This raises the question of whether the offspring of smoking mothers may have a predisposition to neurodegeneration in adulthood.

Activation of TLRs stimulates the production of major inflammatory cytokines IL-1β and IL-6 in monocytes, which in turn enhances the expression of TLRs via a positive feedback loop^38^. In this study, TLR4 mRNA expression was downregulated in the SE mothers’ brains, which is consistent with the reduced expression of IL-1β and IL-6 mRNA we observed. The response of TLR4 expression to cigarette smoke has been found to differ between tissues. A thirty minute exposure to cigarette smoke increased TLR4 mRNA expression in gingival epithelial cells^39^; while TLR4 mRNA expression was reduced in human macrophages and primary monocytes after treatment with cigarette smoke extract^40^. However, cigarette smoking is often associated with increased inflammatory cytokines such as TNFα, IL-1β and IL-6 in the blood and organs, which are also regulated by EGR1^41^. Acute nicotine administration can increase the expression of TNFα, IL-1β and IL-6 in rat brains^42^. Khanna and colleagues found that following 30 days of exposure to 4 cigarettes/day in rats, there was a significant increase in brain inflammatory cells^43^. The difference observed in markers of brain inflammation between the Khanna study and our study may be due to two reasons. Firstly 3R4F research grade cigarettes were used in Khanna’s study, which can contain different chemicals from the commercial cigarettes consumed by the humans in this study. Secondly, nicotine and cotinine clearance is known to increase during pregnancy, which may reduce the overall impact of cigarette smoke exposure^44^,^45^, although the change in nicotine metabolism during lactation is unclear. This may affect brain inflammatory response to nicotine and most importantly other chemicals in the cigarette smoke. In addition, in another study, three weeks of treatment with low dose nicotine (<0.5 cigarette/day) was able to reduce inflammatory gene expression in the rat brain^46^. In the current study, we found that the blood cotinine levels in SE mothers were equivalent to 1–2 cigarettes/day in humans^47^. Thus, our effect may be more comparable to the low-dose nicotine treatment previously demonstrated in the literature^46^, which is consistent with our observation of reduced brain expression of inflammatory markers in SE mothers. However, increased brain oxidative stress in the SE dams was also observed increased in the study by Khanna *et al*.^43^, suggesting cigarette smoke is a direct cause of oxidative stress regardless of the other responses.

Cotinine levels in P20 offspring in this study are similar to those reported in human infants of continuous smokers^48^, where chemicals in cigarettes were delivered through the breast milk. Interestingly, the changes of brain inflammatory markers in the SE offspring were somewhat different from their mothers. Only TLR4 mRNA expression at P1 was similar to the SE mothers, which is consistent with a previous study where reduced TLR4 in cord blood was observed in the neonates of smoking mothers^39^. However, TNF-α mRNA was reduced in P1 offspring. This suggests a differential impact of cigarette smoke exposure on mothers and chemicals delivered through the cord blood to their offspring *in utero*. This is not surprising as blood nicotine concentration in the fetus is normally higher than in the maternal blood. The different levels of nicotine and potentially other chemicals from cigarettes might be likely to account for the different inflammatory response observed in offspring versus the smoking mothers^44^,^45^. Although IL-1β mRNA levels were unchanged in the SE offspring, the persistent increase in IL-1R mRNA observed from birth to adulthood is likely to enhance the inflammatory activity of IL-1β. Surprisingly, at 13 weeks, expression of TLR4, IL-6, and IL-1R mRNA in brain were all increased in SE offspring, which is in contrast to pups at P1 and their SE mothers. This suggests a sustained effect of maternal cigarette smoking in the offspring to change brain inflammatory cytokine production. Microglial activation is known to be increased by low-dose cigarette exposure SE (plasma cotinine levels of 10 ng/ml) in mice^49^, which may be the reason for increased inflammatory cytokine expression that we observed in the P20 SE offspring. The increase in the inflammatory cytokines at 13 weeks may render them more susceptible to the development of neurodegenerative diseases. Neuroinflammation has been shown to plays a crucial role in the development of neurodegeneration. Rodent studies have shown that smoking can lead to pathological changes and accelerated progression of aging^50^,^51^. In murine cortical neurons, an increase in TLR4 can lead to β-amyloid-induced apoptosis through jun N-terminal kinase – and caspase-3-dependent mechanisms^52^. Injection of IL-1 into rat brain can lead to an elevation of β-amyloid^53^, which has been shown to play a role in Alzheimer’s Disease (AD). Overexpression of cytokines such as IL-6 can have a neurotoxic effect that leads to neurodegenerative disorders in some individuals^54^. The elevation of TLR4, IL-1R and IL-6 in adult SE offspring suggests that they might be more vulnerable to diseases such as AD. The incidence of AD is higher among smokers^55^, which may be transmitted to the offspring due to brain changes by intrauterine SE as we have shown here. However, post-injury functional recovery of neurons is also IL-6 dependent, due to its role in neuronal and glial regeneration^56^,^57^,^58^. In IL-6 knockout mice there was a 60% reduction in neuronal density and decreased sensory function after injury^59^. Therefore decreased IL-6 mRNA in SE dams might also indicate a compromised ability for recovery when brain injury occurs, which requires further investigation.

Although smoking itself is not considered to be able to cause hypoxia in the brain, maternal smoking is one of the risk factors for intrauterine hypoxia, which can lead to sudden infant death after birth^60^. This is mainly due to the restriction of placental blood flow caused by nicotine, which can reduce not only nutrients, but also oxygen supply to the growing foetus^61^. Under normoxic conditions, HIF-1α protein is tightly regulated by oxygen levels. It is maintained at a low level through continuous degradation by the ubiquitin-proteosome pathway^62^. However, long-term hypoxia and the activation of various signal transduction pathways can prevent HIF-1α degradation^62^. The expression of HIF-1α protein is organ specific under systemic hypoxia^63^, where HIF-1α binds to the promoter of TLR4 to upregulate TLR4 expression^64^. HIF-1α can also initiate various other hypoxia-inducible adaptations by regulating glycolysis, erythropoiesis, angiogenesis and cell proliferation^65^. Smoking itself has previously been shown to inhibit hypoxia-inducible adaptations in peripheral tissues^66^. Cigarette smoke exposure SE can also impair the production of HIF-1α as well as the stabilization of HIF-1α protein levels^66^. HIF-1 has been shown to have complex roles in the brain following injury and depending upon the stimulus and cell type being examined, can be neurotoxic or neuroprotective^67^,^68^. Hypoxia-induced angiogenesis is suggested to be inhibited in the smokers due to an impairment in the HIF-1 pathway^66^, thus smokers are more likely to suffer from more severe injury during stroke, with a worse prognosis compared with the non-smokers^69^. Here we only observed marginal reduction in brain HIF-1α protein in the SE mothers, which may be due to the low-dose and the relatively short exposure to cigarette smoke. EGR1 regulates the expression of HIF-1α during hypoxia^70^. Although EGR1 protein was not changed in the SE mothers, it was reduced in the newborn SE offspring. This may be due to a direct suppression by chemicals in the cigarette smoke inhaled by the mothers, which are at higher levels in newly born offspring compared with the mothers. It also needs to be noted that EGR1 is not the only regulator of HIF-1α, therefore the unchanged brain HIF-1α levels we observed in P1 and P20 offspring exposed to cigarette smoke SE may be due to the actions of other factors that regulate HIF-1 function, which is beyond the scope of this study. After birth, brain oxygen is replenished, while the impact of cigarette smoke components in the breast milk on HIF-1α levels also disappeared after weaning. This may lead to higher brain HIF-1α levels at adulthood. However, HIF-1α itself can induce inflammatory responses in the brain^71^ as we have observed in the adult SE offspring where TLR4 and TNFα are both upregulated. As brain EGR1 levels were unchanged at this age, it may not play a major role in increasing HIF-1α in the SE offspring. Considering the protective effect of HIF-1α, its increase in the brains of SE offspring may be an adaptation to protect against increased oxidative stress in the brain. It has been suggested that under normoxic conditions, increased oxidative stress due to excessive mitochondrial ROS production can increase HIF-1α protein levels^72^. This impact of oxidative stress is also seen in the brain of SE offspring here. Under basal conditions, 90% of ROS are produced in the mitochondria, mainly by OXPHOS complexes I and III in the electron transport chain^73^. Complex II is involved in the conversion of metabolic intermediates to complement the action of complexes I and III^74^. When the activities of both complexes I and III are inhibited, complex II will generate large amounts of superoxide^75^. Complex IV (known as cytochrome oxidase) is a crucial regulator for OXPHOS, the dysfunction of which leads to reduced ATP levels^76^; while Complex V converts ADP to ATP^74^. ROS generated during OXPHOS is both beneficial and detrimental to the cells^77^. It can activate the antioxidant defence network to prevent damage to the host itself. Thus, ROS are tightly regulated by antioxidant enzymes such as MnSOD^78^. In the SE offspring, MnSOD was unchanged at P1 and P20, possibly due to the protective effect of the antioxidant-rich breast milk^79^. Changes in mitochondrial OXPHOS complexes in brains of SE offspring mirror the changes of TOM20 levels, both at weaning and in adulthood. TOM20 imports protein into the mitochondria from the outer mitochondrial membrane^80^, reflecting changes in energy needs by the mitochondria and body. Reduced OXPHOS complex and TOM20 levels at P20 may be due to a redistribution of nutrients after birth required for the catch-up growth of the other organ systems commonly seen in offspring from smokers. Similar to their mothers, brain mitochondrial complexes I–V and TOM20 in the SE offspring were all increased at 13 weeks, suggesting increased substrate metabolism, which can result in increased ROS production. Conversely, mitochondrial MnSOD levels are low and may not be sufficient to clear excess ROS, resulting in oxidative stress and related tissue damage. Here, we have observed increased levels of nitrotyrosine protein in the brains of SE offspring at 13 weeks. Elevated nitrotyrosine levels are harmful to the brain, and is one factor contributing to neurodegenerative diseases in humans^81^. However, the link between increased brain oxidative stress and any brain dysfunction in the SE offspring remains to be elucidated.

In conclusion, maternal cigarette SE differentially changed brain inflammatory and hypoxia response markers in the mother and offspring. However, oxidative stress and mitochondrial damage were changed in a similar manner in both SE mothers and their offspring, which may predispose them to neurodegeneration in later life.

## Additional Information

**How to cite this article**: Chan, Y. L. *et al*. Impact of maternal cigarette smoke exposure on brain inflammation and oxidative stress in male mice offspring. *Sci. Rep*. **6**, 25881; doi: 10.1038/srep25881 (2016).

## Supplementary Material

Supplementary Information

## Figures and Tables

**Table 1:** TaqMan probe sequence (Life Technologies, CA, USA) for rt-PCR.

Gene	NCBI gene references	Probe Sequence	ID
EGR1	NM_007913.5,M20157.1,M19643.1	TGAGCACCTGACCACAGAGTCCTTT	Mm00656724_m1
HIF-1α	NM_010431.1,AF003695.1,X95580.1	CAGCAGGAATTGGAACATTATTGCA	Mm00468878_m1
IL-1β	NM_008361.3,M15131.1,BC011437.1	TCCTTGTGCAAGTGTCTGAAGCAGC	Mm01336189_m1
IL-1R	NM_001123382.1,NM_008362.2,M20658.1	AGCTGACCCAGGATCAATGATACAA	Mm00434237_m1
IL-6	NM_031168.1,X06203.1,X54542.1	ATGAGAAAAGAGTTGTGCAATGGCA	Mm00446190_m1
TLR4	NM_021297.2,AF095353.1,AF110133.1	CCCTGCATAGAGGTAGTTCCTAATA	Mm00445273_m1
TNFα	NM_013693.2,X02611.1,M13049.1	CCCTCACACTCAGATCATCTTCTCA	Mm00443259_g1

**Table 2:** Parameters of the male offspring at different ages.

	Day 1		Day 20		Week 13	
Offspring	SHAM	SE	SHAM	SE	SHAM	SE
	n = 13	n = 15	n = 17	n = 18	n = 15	n = 11
Body weight (g)	1.86 ± 0.11	1.47 ± 0.04	9.9 ± 0.22	9.7 ± 0.22	26.8 ± 0.5	25.5 ± 0.3**
Brain (mg)	7.9 ± 0.19	5.8 ± 0.3**	18 ± 1.1	20 ± 1.6	30.6 ± 0.2	29.8 ± 0.2**
Brain% of body weight	4.3 ± 0.2	4 ± 0.2	1.8 ± 0.1	2.0 ± 0.1	1.1 ± 0.01	1.2 ± 0.01

Results are expressed as mean ± S.E.M. Data were analysed by student’s unpaired t test.

**p < 0.01, compared with the SHAM offspring at the same age.
