# Supplementary material for: Impact of maternal cigarette smoke exposure on brain inflammation and oxidative stress in male mice offspring
Source: Sci Rep. 2016 May 12;6:25881. doi: 10.1038/srep25881 (PMC4864383; doi:10.1038/srep25881)
Supplement: Supplementary Information [file srep25881-s1.pdf]

Supplementary Information

Impact of maternal cigarette smoke exposure on brain inflammation and oxidative stress in male mice offspring

Yik Lung Chan[1], Sonia Saad[2], Carol Pollock[2], Brian Oliver[1], Ibrahim Al-Odat[1], Amgad A. Zaky[2], Nicole Jones[3], Hui Chen[1*]

Supplementary Figure 1. Representative whole gel images of brain MnSOD (a), TOM20 (b), and OXPHOS complexes (CI, CII, CIII, CIV and CV) (c) in the SHAM and SE dams. MnSOD: manganese superoxide dismutase; OXPHOS: oxidative phosphorylation; SE: smoke exposed; TOM20: translocase of the mitochondrial outer membrane.

Supplementary Figure 2. Representative whole gel image of brain HIF-1α (a) and EGR1 (b) in the SHAM and SE dams. EGR1: early growth response factor: HIF-1α: hypoxia-inducible factor ; SE: smoke exposed.

Supplementary Figure 3. Representative whole gel image of brain MnSOD (a), TOM20 (b), and OXPHOS complexes (CI, CII, CIII, CIV and CV) (c) in the brain mitochondria in the offspring of SHAM and SE mothers at different ages. MnSOD: manganese superoxide dismutase; OXPHOS: oxidative phosphorylation; SE: smoke exposed; TOM20: translocase of the mitochondrial outer membrane.

Supplementary Figure 4. Representative whole gel image of brain hypoxia markers in the offspring of SHAM and SE mothers at different ages (a-f). EGR1: early growth response factor: HIF-1α: hypoxia-inducible factor ; SE: smoke exposed.

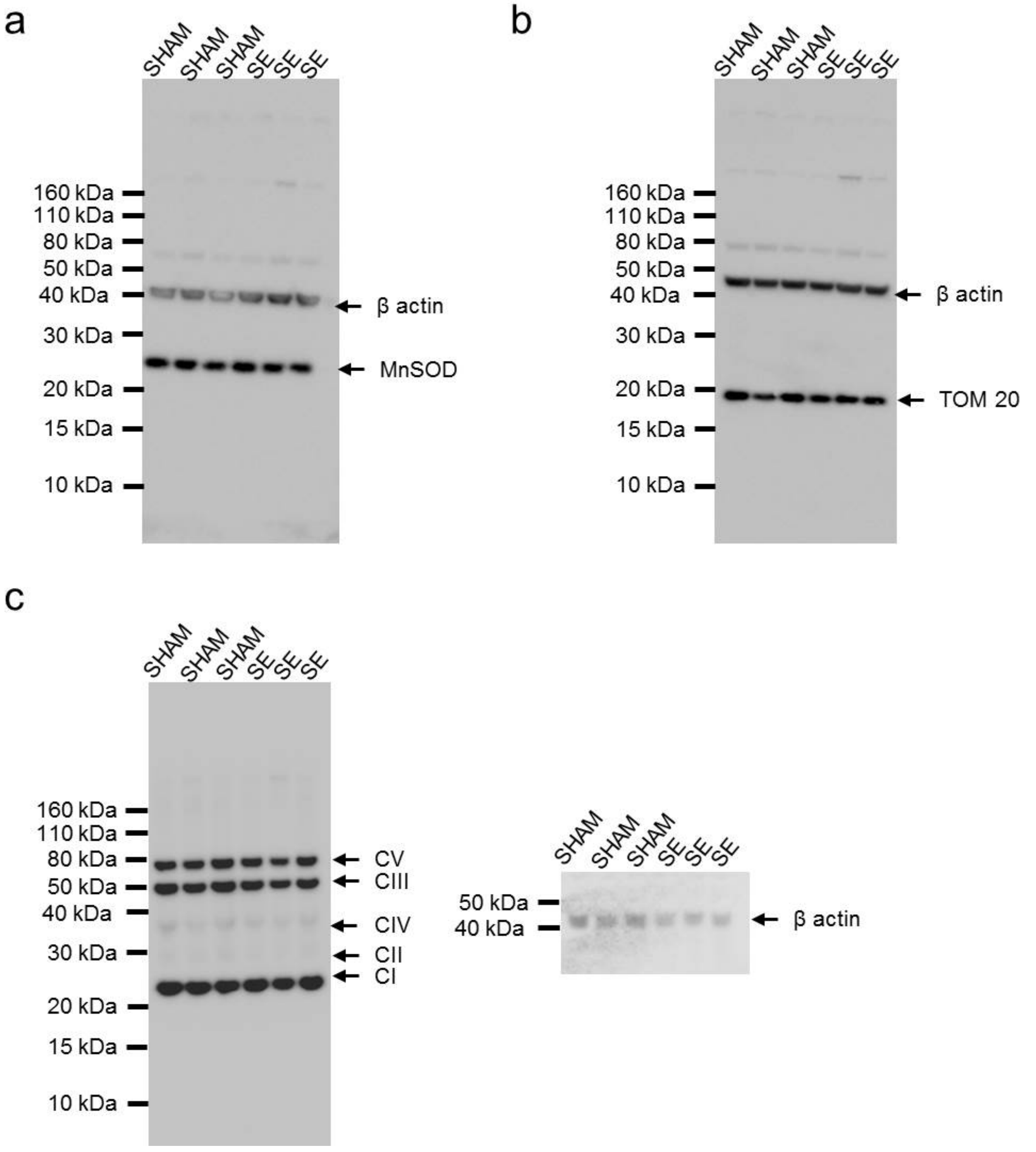

Supplementary Figure 1.

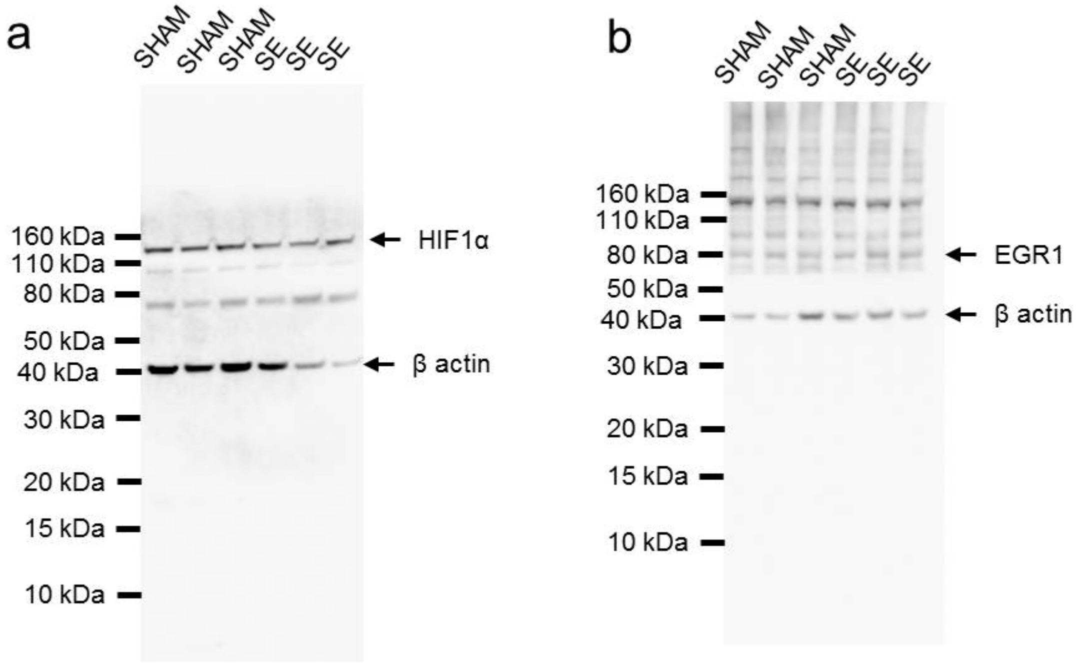

Supplementary Figure 2.

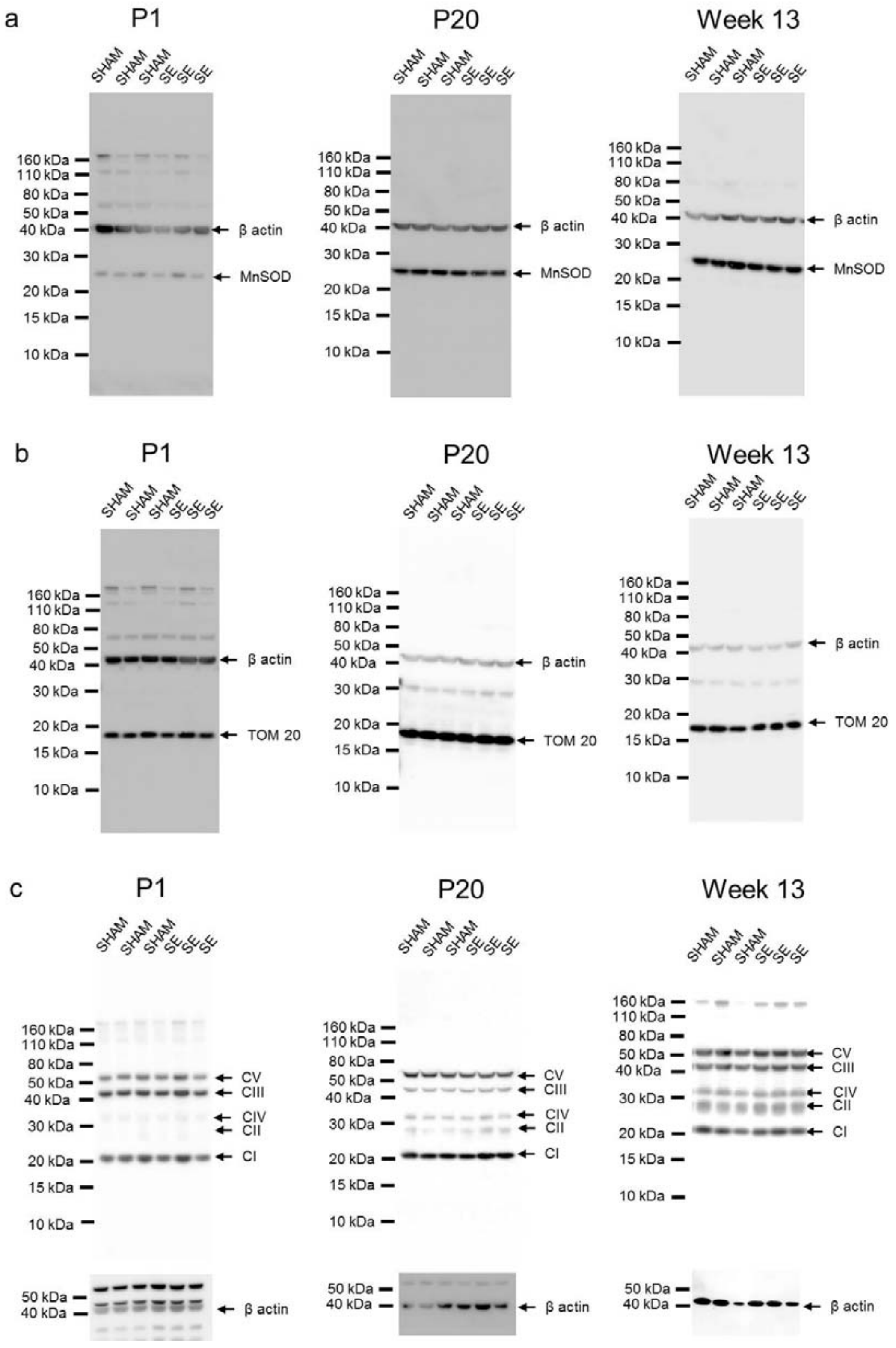

Supplementary Figure 3.

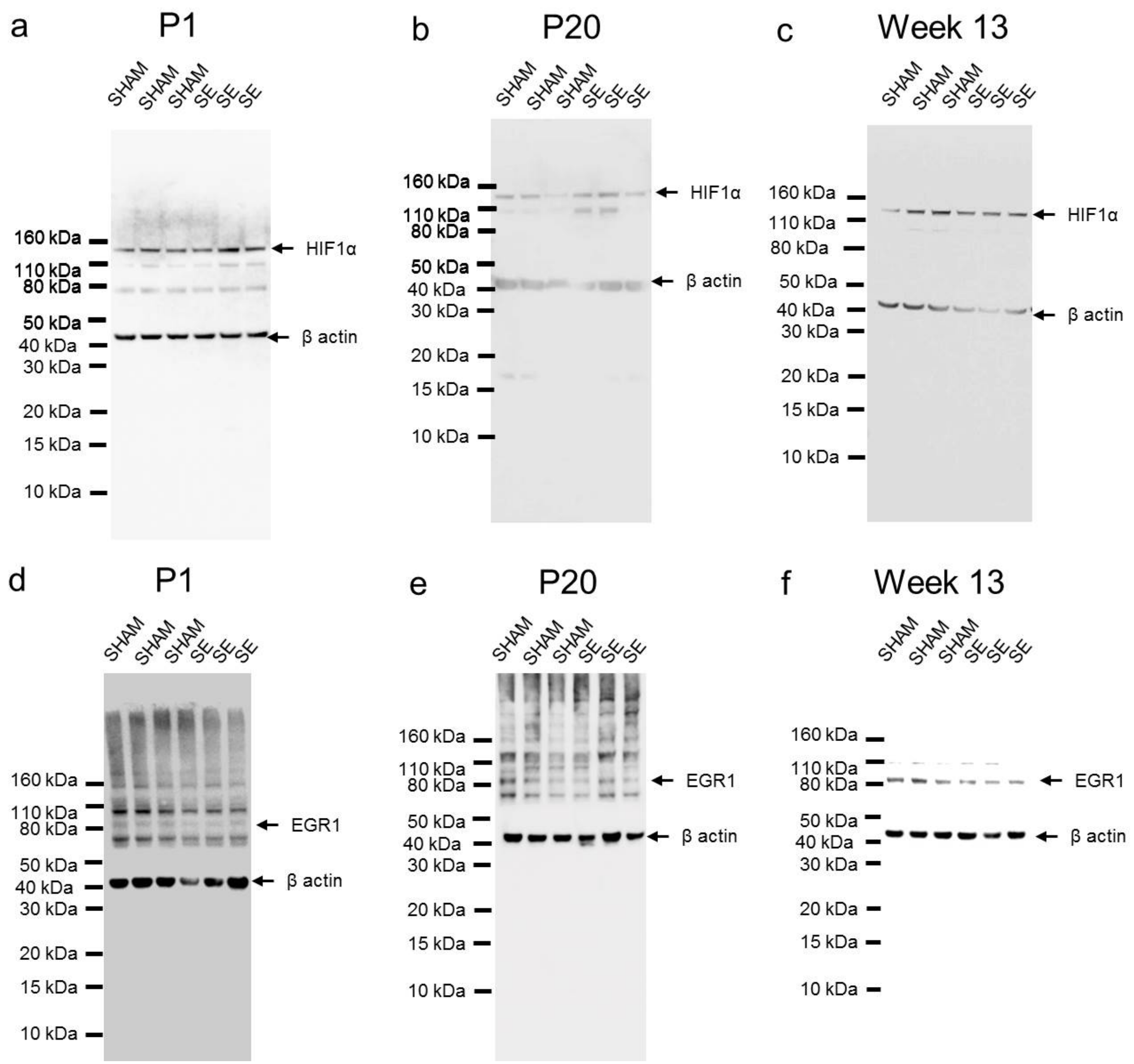

Supplementary Figure 4.
